# Accelerated and navigator-gated look-locker imaging for cardiac T1 Estimation (ANGIE) with reduced motion artifact

**DOI:** 10.1186/1532-429X-14-S1-O110

**Published:** 2012-02-01

**Authors:** Bhairav B Mehta, Xiao Chen, Michael Salerno, Christopher M Kramer, Frederick H Epstein

**Affiliations:** 1 Department of Biomedical Engineering, University of Virginia, Charlottesville, VA, USA; 2 Department of Radoilogy, University of Virginia, Charlottesville, VA, USA; 3 Department of Medicine, Cardiology Division University of Virginia, Charlottesville, VA, USA

## Background

T1 mapping of the left ventricle is routinely performed using Modified Look-Locker Inversion Recovery (MOLLI) [1] in a variety of disease settings [2]. MOLLI requires relatively long breathholds, and consequently sequences of MOLLI images may be misregistered due to respiratory drift. We sought to develop a T1 mapping sequence that would not require breathholding and would not have misregistration problems. We used k-space segmentation and navigator gating to eliminate respiratory artifact, and compressed sensing [3] (CS) to accelerate image acquisition. CS utilizes undersampling in k-t space along with nonlinear iterative image reconstruction. CS is well-suited to Look-Locker imaging because these images, which vary smoothly from one inversion time to the next, exhibit time-domain sparsity.

## Methods

Imaging was performed on a 1.5T scanner (Avanto, Siemens, Germany). The proposed sequence, Accelerated and Navigator-Gated look-locker Imaging for cardiac T1 Estimation (ANGIE), was compared to a standard MOLLI sequence in 6 healthy volunteers (age 25±2 yrs). ANGIE used inversion-recovery Look-Locker imaging where image acquisition was segmented and accepted or rejected based on navigator gating. Each inversion was followed by 4 consecutive R-R intervals in which segmented data were acquired and 2 R-R intervals for unperturbed T1 relaxation. This inversion and acquisition pattern was repeated until sufficient data for CS reconstruction were acquired. The CS acceleration rate was approximately 2.3. For each frame, we acquired 18 central k-space lines, and the remaining acquired lines were randomly distributed with uniform probability. Other parameters included: resolution=1.4-1.7mm^2^, matrix size=208x144, number of inversion times=12, and number of lines per segment=36. CS reconstruction was implemented offline in MATLAB. Contours were drawn to delineate myocardium on a single image, and these contours were propagated to images at other inversion times. T1 maps were estimated using a three parameter fit^1^. The Dice Similarity Coefficient (DSC) was used to quantify image registration on a scale of 0 (complete misregistration) to 1 (perfect registration) [4].

## Results

**Figure 1:**
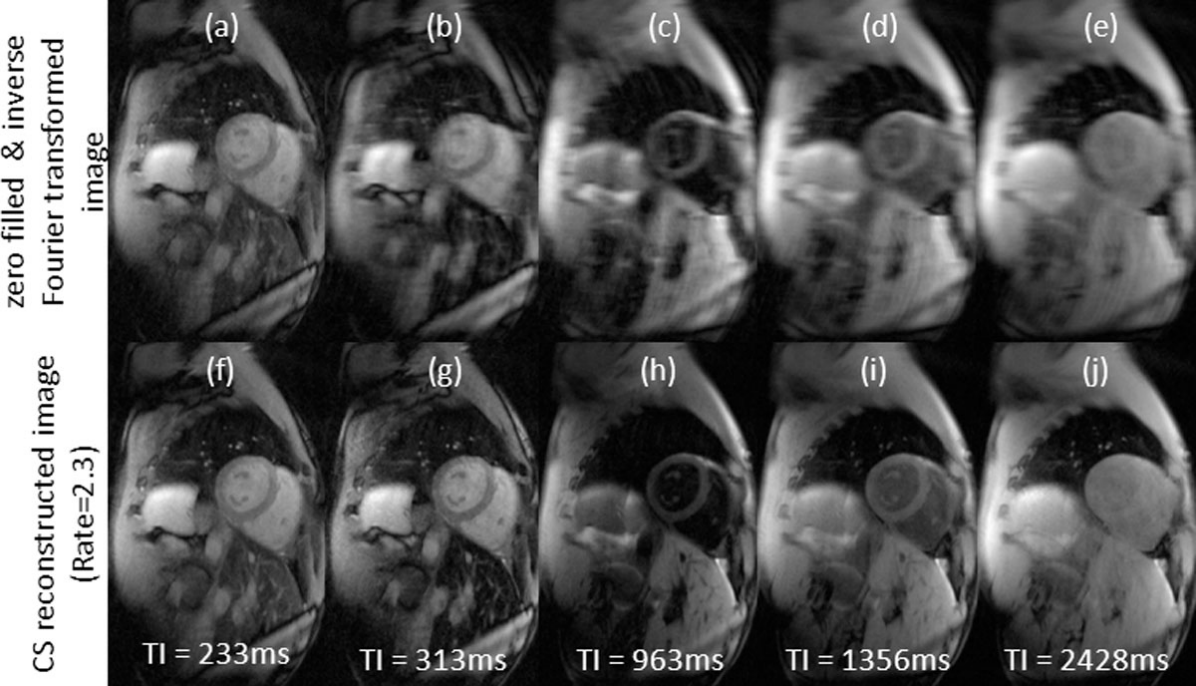
Example of compressed sensing reconstruction of ANGIE images. Five of twelve images at different inversion times are shown. Top row (a-e): images reconstructed by zero filling and inverse Fourier transform. Bottom row (f-j): images reconstructed using compressed sensing. The CS reconstructed images show reduction in aliasing artifacts and increase in image resolution.

Figure 1 (bottom row) illustrates that CS-reconstructed ANGIE images achieved excellent image quality. T1 values, DSC, and scan times for MOLLI and ANGIE are provided in Table 1.

**Table 1:** Comparison of results between MOLLI and ANGIE. Dice Similarity Coefficient (DSC), heart beat (hb)

	MOLLI	ANGIE
T1 (ms)	990 ± 117	941 ± 94
DSC	0.78 ± 0.10	0.89 ± 0.02
Scan Time	17 (hb)	81 ± 28 (s)
Accel. Rate	1.7 ± 0 (Parallel)	2.3 ± 0.4 (CS)

## Conclusions

ANGIE improves registration of the sequence of Look-Locker images compared to MOLLI and accurately measures myocardial T1 in volunteers. CS accelerates image acquisition and, using approximately rate 2 acceleration, provides a clinically acceptable scan time. Preliminary data suggest that higher acceleration rates will enable scan times under one minute per slice in the future, and longer acquisitions may enable higher resolution T1 mapping that could be applied to thinner structures such as the left atrium.

## Funding

This work was funded by Siemens Medical Solutions and NIH R01 EB 001763.

## References

[B1] Messroghli MRM 2004

[B2] Mewton JACC 2011

[B3] Lustig MRM 2007

[B4] Dice Ecology 1945

